# Detection of drug-sensitizing EGFR exon 19 deletion mutations in salivary gland carcinoma

**DOI:** 10.1038/sj.bjc.6604430

**Published:** 2008-06-10

**Authors:** R Dahse, H Kosmehl

**Affiliations:** 1 HELIOS Clinics Erfurt, Institute of Pathology, Nordhauser Street 74, Erfurt 99089, Germany

**Keywords:** allele-specific PCR, epidermal growth factor receptor, mutation screening, salivary adenoid cystic carcinoma, salivary glands

## Abstract

Activating mutations within the epidermal growth factor receptor (EGFR) identify lung adenocarcinoma patients with improved clinical responses to tyrosine kinase inhibitors gefitinib and erlotinib. By screening salivary gland carcinoma, two drug-sensitizing EGFR exon 19 delE746-A750 mutations were identified in an adenocystic and in a mucoepidermoid carcinoma of the parotid gland.

Recent studies (Lynch *et al*, 2004; Paez *et al*, 2004; Eberhard *et al*, 2005; Riely *et al*, 2006) have identified a subset of lung cancer patients with rapid and durable clinical responses to epidermal growth factor receptor (EGFR) tyrosine kinase inhibitors (i.e. gefitinib, erlotinib) and found an underlying association between activating mutations in the EGFR tyrosine kinase domain and therapy response. Two mutations account for approximately 90% of EGFR mutations reported to date in lung adenocarcinoma (Pan *et al*, 2005). An in-frame deletion of 9–24 nucleotides in exon 19 centred around codons 746–750 makes up about 50% of mutations. The second most common mutation is a point mutation at nucleotide 2573 (C**T**G to C**G**G) in exon 21 that results in the substitution of leucine by arginine at codon 858 (L858R).

Organotypical salivary gland carcinomas (SGCs) have a comparable embryonal tissue origin to lung adenocarcinoma. Epidermal growth factor receptor has been shown to be overexpressed in both salivary adenoid cystic carcinoma (ACC) and non-ACC (Agulnik *et al*, 2007). We hypothesised that EGFR-activating mutations in SGC might occur, making EGFR a potential molecular target for therapy also in these rare tumours with poor prognosis and limited response to traditional chemotherapies.

## Materials and methods

### Samples and DNA isolation

Formalin-fixed tumour samples were obtained from 25 patients (16 males and 9 females with a median age at diagnosis of 61 years) treated at the HELIOS Clinical Center Erfurt, Germany, with the histopathological diagnosis of a SGC according to the WHO classification (Barnes *et al*, 2005). The study cohort consisted of ACC (*n*=11), mucoepidermoid carcinoma (*n*=6), epithelial–myoepithelial carcinoma (*n*=6) and acinic cell carcinoma (*n*=2). This study was conducted in accordance with the principles of the Declaration of Helsinki, as adopted by the 29th World Medical Assembly, Helsinki, Finland.

Heterozygous mutant control DNA was extracted from lung cancer cell lines NCI-H-1650 and NCI-H-1975 (American Type Culture Collection, LGC Promochem, Wesel, Germany), which contain the exon 19 E746_A750 deletion and the exon 21 L 858R point mutation, respectively.

DNA was isolated from microdissected tumour areas using the QIAamp DNA Mini Kit (Qiagen GmbH, Hilden, Germany).

### Exon 19 E746_A750del and exon 21 L 858R point mutation detection assays

Mutation screening was performed by two allele-specific PCR assays and discrimination of the allele-specific PCR fragments by agarose gel electrophoresis.

For detecting the exon 19 deletion, a so-called Bi-PASA assay was used with allele-specific primers, which can detect both possible nucleotide deletions (c.2235_2249del15 and c.2236_2250del15) that give rise to an E746_A750del protein change. Exon 21L 858R was screened by allele-specific PCR (Dahse *et al*, 2008).

### Sequencing

The EGFR mutations were confirmed by genomic DNA sequencing of the relevant regions of exon 19. PCR products were purified with the MinElute PCR Purification Kit (Qiagen GmbH). Genomic sequencing (MWG Biotech, Martinsried, Germany) was performed using the appropriate downstream PCR primer.

## Results

DNA was isolated from microdissected areas of 25 SGCs (obtained by biopsy or surgical resection) and screened for the both most common EGFR-activating mutations.

The mutant exon 21 2573G allele was not detectable.

**Figure 1:**
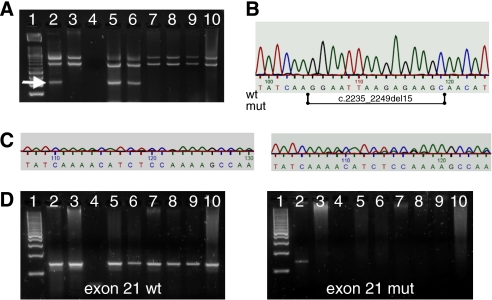
Epidermal growth factor receptor (EGFR) mutation screening in malignant salivary gland tumours. (**A**) Exon 19 E746_A750 deletion Bi-PASA assay. The Exon 19 deletion is detected by an allele-specific 134 bp fragment (arrow). It is seen in the positive control (NCI-H-1650, lane 2) and was found in a mucoepidermoid carcinoma of the left parotid gland (lane 5) and in an adenoid cystic carcinoma (ACC) of solid type of the right parotid gland (lane 6). No deletions were detected in other malignant tumours (lane 7: ACC; lanes 8 and 9: mucoepidermoid carcinoma; lane 10: acinic cell carcinoma). Lane 1: molecular weight marker; lane 3: wild-type DNA control, lane 4: no template PCR control (water). (**B**) Part of the wild-type (wt) EGFR exon 2 genomic sequence. The 15 bp deletion is schematically depicted (mut). (**C**) Genomic sequencing of EGFR exon 2 in both SGC tumour samples harbouring the deletion c.2235_2249del15. (**D**) Exon 21 L858R allele-specific PCR assay. Left figure: the wild-type-specific PCR fragment was detected in all tumour samples (lanes 5–10), in the heterozygous positive control (NCI-H-1975, lane 2) and in the wild-type DNA control (lane 3). Right figure: the mutant G 2573G allele is amplified only in the positive control and was not found in the tumour samples.

We identified two exon 19 E746_A750 deletions. Genomic sequencing confirmed both exon 19 deletions to be the variant c.2235_2249del15 (Figure 1). Case 1 was a 54-year-old female patient with a mucoepidermoid carcinoma of the left parotid gland grade 2, case 2 was a 69-year-old male patient with an ACC of solid type of the right parotid gland.

The exon 19 Bi-PASA assay included a PCR control fragment that was always amplified indicating the integrity of the isolated DNA from microdissected clinical tissue samples.

## Discussion

This is the first report of the detection of an EGFR hot-spot mutation (known from lung cancer to be correlated to gefitinib/erlotinib therapy sensitivity) in SGCs.

Approximately 10–30% of lung carcinoma contain EGFR mutations (Yatabe and Mitsudomi, 2007). Retrospective studies found that after treatment with gefitinib or erlotinib, lung cancer patients with EGFR exon 19 deletions had a longer overall survival when compared with patients with EGFR L858R (Jackman *et al*, 2006; Riely *et al*, 2006).

It is known that EGFR mutations in the tyrosine kinase domain are seldom acquired in the cancers of other organs (EGFR mutation database: http://www.cityofhope.org/cmdl/egfr%5Fdb/).

We detected in 2 out of 25 SGC (8%/95% exact confidence limit: 1.4–27.5%) the recurrent delE746-A750 mutation known from lung cancer.

Salivary gland carcinomas are treated mainly with surgery and radiation, and response rates to conventional chemotherapy are generally low. Given this, patients with SGC might profit from trials of investigational new therapies. C-kit is overexpressed in a wide percentage of SGCs, but clinical trials with single-agent imatinib have been negative. ErbB1 and ErbB2 are also frequently overexpressed in salivary gland cancers and this has provided the rationale for clinical trials with trastuzumab, cetuximab, gefitinib and lapatinib. Twenty-nine patients with incurable SGCs were accrued in a phase II trial of gefitinib, an orally active ErbB1-inhibitor. Gefitinib was associated with a 53% stable disease rate (10/19) in ACC, which was maintained for 16 weeks in 26% (5/19) of the patients in this cohort. Cetuximab (Erbitux, C225), a human-murine chimeric monoclonal antibody to ErbB1, has been tested in a phase II study in 30 patients with recurrent and/or metastatic SGCs. Among 22 patients evaluable for response after at least 3 months of treatment, 11 patients had stable disease, 9 had progressive disease, while 2 patients refused to continue after 1 month (Milano *et al*, 2007). In a phase II study of lapatinib in EGFR and/or erbB2-expressing SGCs, the antitumour effects appeared mainly cytostatic (Agulnik *et al*, 2007).

In conclusion, the presence of EGFR-activating mutations in SGCs might identify patients that would profit from tyrosine kinase inhibitor therapies. On the other hand, those patients with an EGFR-activating mutation would not benefit from the competitive inhibition of EGF on its receptor. The identification of EGFR-activating mutations is essential for the selection of the EGFR-targeting therapy strategy (receptor-specific antibody *vs* tyrosine kinase inhibitor). Because the growth factor signal is generated in the mutated receptor itself, a prereceptor blocking of the ligand has no specific effect. A maximal effect is yielded by blocking the signalling pathways downstream of the receptor.

Our finding of the presence of activating EGFR mutations in SGCs should initiate further studies on other more rare kinase domain mutations that are known to be associated with drug sensitivity (G719A/C point mutation in exon 18 and L861Q in exon 21).
